# From Nutritional Profile to Circular Bioeconomy: A Review of Sea Buckthorn Oil and By-Product Valorization

**DOI:** 10.3390/foods15111873

**Published:** 2026-05-25

**Authors:** Xiaojing Jiang, Menghuan Sun, Wenqi Deng, Min Zhu, Liang Wang, Li Zheng, Jun Xing, Jingyang Hong

**Affiliations:** College of Smart Agriculture, Xinjiang University, Urumqi 830011, China; jiangxiaojing@stu.xju.edu.cn (X.J.); 107552403518@stu.xju.edu.cn (M.S.); 18690996392@163.com (W.D.); minna0130@163.com (M.Z.); wl1390593786@163.com (L.W.); xjdxzl01@163.com (L.Z.); xinjung@163.com (J.X.)

**Keywords:** *Hippophaë rhamnoides* L., sea buckthorn oil, bioactive compounds, polyunsaturated fatty acids, antioxidant activity, by-product valorization, circular bioeconomy

## Abstract

Background: This review summarizes the current knowledge on the composition, bioactive constituents, health-related effects, and by-product utilization of sea buckthorn (*Hippophaë rhamnoides* L.) seed and pulp oils. Review approach: This review covers studies on fatty acid composition, minor bioactive compounds, antioxidant and anti-inflammatory activities, lipid metabolism-related effects, and the valorization of processing by-products, with evidence primarily derived from in vitro and in vivo studies. Results: Sea buckthorn produces two distinct oils: seed oil, characterized by high levels of polyunsaturated fatty acids, tocopherols, and phytosterols, and pulp oil, which is rich in palmitoleic acid and carotenoids. These compositional differences contribute to their antioxidant, anti-inflammatory, and lipid-regulating activities. In addition, the utilization of by-products, particularly polyphenol- and fiber-rich residues, has gained increasing attention for improving resource efficiency and sustainability of the industry. Conclusions: Sea buckthorn oil is a promising source of functional lipids and bioactive compounds. However, current evidence is largely based on experimental studies, and further research is needed to clarify the mechanisms of action, bioavailability, dose–response relationships, and clinical efficacy. Advances in green extraction technologies and integrated utilization strategies may further support the sustainable development of sea buckthorn resources.

## 1. Introduction

**Figure 1:**
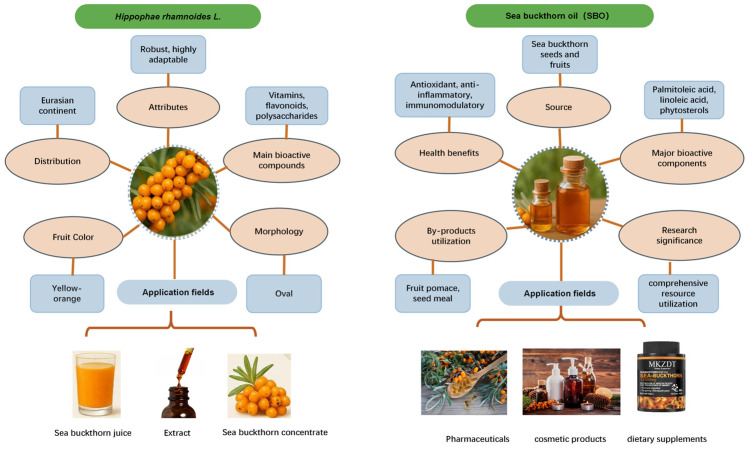
Functional distinctions between sea buckthorn fruit and SBO.

Sea buckthorn (*Hippophae rhamnoides* L.) is a hardy shrub widely distributed across Eurasia and is recognized as an important functional plant resource. The plant exhibits strong tolerance to harsh environmental conditions and has been extensively cultivated for nutritional and medicinal applications [1]. According to the International Sea Buckthorn Association, global cultivation covers approximately 35 million acres, with China accounting for nearly 88% of the total area, primarily in the northwestern, northern, and northeastern regions [2]. The fruits are rich in bioactive compounds, including L-ascorbic acid, flavonoids, polysaccharides, phytosterols, tocopherols, fatty acids, and carotenoids, which contribute to their significant nutritional and pharmacological values [3]. An overview of the botanical characteristics, oil sources, bioactive composition, health benefits, and by-product utilization of sea buckthorn is presented in Figure 1.

Sea buckthorn fruit yields two chemically distinct oil fractions—seed oil (SBSO) and pulp oil (SBPO)—each possessing uniquely divergent fatty acid and bioactive profiles. Specifically, SBSO is predominantly composed of polyunsaturated fatty acids, namely linoleic (ω-6) and α-linolenic (ω-3) acids, whereas SBPO is characterized by an abundance of palmitoleic acid (ω-7) and lipophilic carotenoids [4]. This fundamental compositional dichotomy dictates their respective physiological efficacies, ranging from antioxidant and anti-inflammatory activities to lipid metabolic regulation and tissue regeneration [5,6]. Nevertheless, despite surging scientific interest in sea buckthorn oil (SBO), current research remains largely fragmented. Existing literature frequently investigates isolated bioactives or localized biological effects, leaving a critical gap in the systematic and comparative evaluation of the distinct compositional and functional properties of SBSO versus SBPO.

Furthermore, although sea buckthorn processing generates substantial volumes of by-products rich in residual oil, polyphenols, and dietary fiber, their potential for high-value valorization remains largely underexploited. Current challenges include a lack of standardization in extraction methods, a limited mechanistic understanding of bioactive interactions, and insufficient clinical evidence to substantiate health claims. Existing reviews have primarily emphasized either phytochemical composition or pharmacological activities, while integrated analyses linking oil composition, biological function, and by-product valorization remain scarce.

Given the diverse functional properties of both the extracted oils and their residual matrices, a comprehensive synthesis of current research is essential for advancing sustainable resource development. Therefore, this review provides a critical and integrated overview of SBO, with particular emphasis on delineating the comparative characteristics of SBSO and SBPO. Ultimately, this work aims to systematically summarize the existing literature regarding their chemical compositions, therapeutic applications, and the high-value utilization of SBO by-products.

## 2. Chemical Composition of SBO

**Figure 2:**
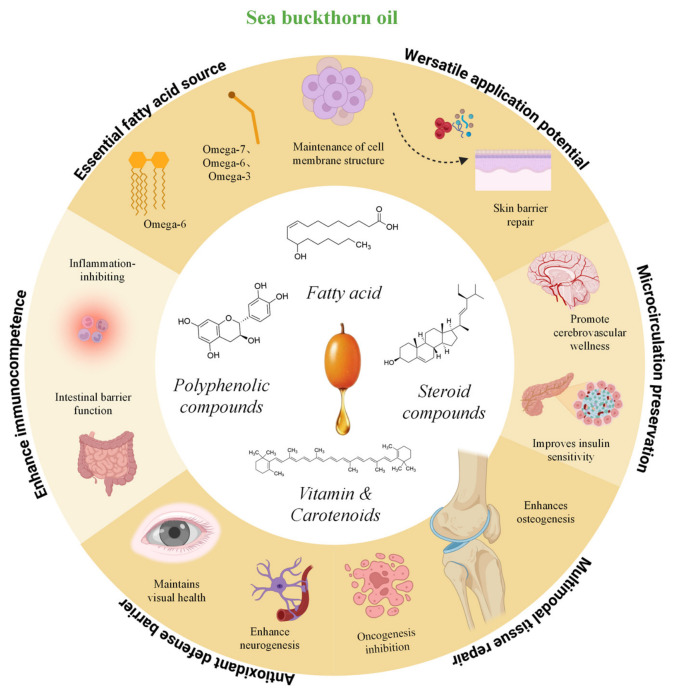
Therapeutic efficacy and health benefits of SBO.

Sea buckthorn yields two distinct oil fractions—seed oil (SBSO) and pulp oil (SBPO)—which differ significantly in their fatty acid profiles and minor bioactive constituents. SBSO is typically extracted from seeds via cold pressing or supercritical CO_2_ extraction and is characterized by a high content of polyunsaturated fatty acids (PUFAs), accounting for approximately 60–70% of the total fatty acids. In contrast, SBPO, derived from the fruit mesocarp and exocarp, contains higher proportions of monounsaturated fatty acids (MUFAs) and carotenoids (Figure 2) [7].

Linoleic acid (C18:2 ω-6, 35–40%) and α-linolenic acid (C18:3 ω-3, 25–30%) are the predominant fatty acids in SBSO, whereas palmitoleic acid (C16:1 ω-7, 30–40%) and palmitic acid (C16:0, 25–30%) predominate in SBPO [8]. Beyond their fatty acid composition, sea buckthorn oil fractions also contain a wide range of minor bioactive constituents, including phytosterols, tocopherols, carotenoids, phenolic compounds, dietary fibers, and residual proteins. However, the concentrations and distribution patterns of these compounds vary considerably between SBSO, SBPO, and processing by-products. To provide a clearer comparison of the major bioactive constituents reported in different fractions, their representative concentration ranges and functional characteristics are summarized in Table 1.

Reported compositional values fluctuate significantly among studies owing to differences in cultivar, geographical origin, fruit maturity, processing conditions, and extraction methodologies. This inherent variability complicates direct comparisons across studies and may confound the interpretation of the reported biological activities of SBO. Therefore, standardized analytical protocols and reporting criteria are imperative to improve data comparability and support robust future functional evaluations and industrial applications.

### 2.1. Fatty Acids

#### 2.1.1. Palmitic Acid

Palmitic acid (PA, C16:0) is the predominant saturated fatty acid in SBSO, accounting for 10–15% of the total fatty acid composition. Although this concentration is notably lower than the 30–35% typically found in tropical oils such as palm or coconut oil, PA remains integral to the unique therapeutic efficacy of SBSO [32]. To preserve this specific profile, cold pressing and supercritical CO2 extraction are utilized, as these methods prevent the thermal degradation of the surrounding bioactive matrix, which defines the functional superiority of SBSO over refined oils.

Within the SBSO matrix, PA serves a dual role: it provides structural stability and modulates specific biological pathways. As a saturated component, PA reinforces membrane rigidity by stabilizing triacylglycerol (TAG) and phospholipid structures, which are essential for maintaining skin barrier integrity. Crucially, in dermatological applications, the PA fraction within SBSO—rather than acting merely as an isolated compound—exhibits distinct anti-inflammatory activity [33,34]. This targeted anti-inflammatory response, characterized by a decrease in pro-inflammatory chemokines and modulated mast cell infiltration, is attributed to the synergistic presence of PA alongside SBSO’s unique phytosterol and tocopherol fractions. Collectively, these components facilitate barrier repair without the adverse effects typically associated with corticosteroids [35].

The bioactivity of PA within SBSO is highly context-dependent. While PA provides an essential structural framework, the high concentrations of linoleic acid (C18:2) and α-linolenic acid (C18:3) primarily drive the oil’s proliferative and regenerative outcomes. Evidence indicates that these PUFAs, when synergistically balanced with the PA content in SBSO, promote keratinocyte and fibroblast proliferation and expedite cellular migration [15]. The specific PA:PUFA ratio inherent to SBSO (approximately 1:4 to 1:5) optimizes membrane fluidity for cellular signaling while maintaining physical barrier density—a biological equilibrium that high-PA oils (such as palm oil) cannot replicate due to their pronounced PUFA deficiency.

This interplay is equally vital for glycemic control in metabolic regulation. Although isolated PA is a poor activator of G-protein-coupled receptor 40 (GPR40), its integration into the SBSO-specific TAG matrix—alongside high-affinity ligands such as linoleic acid and phytosterols—enhances the responsiveness of pancreatic β-cells via the PLC-IP3-calcium signaling pathway [36]. Consequently, PA within SBSO does not act merely as a simple saturated fat; rather, it functions as a structural–functional hub that stabilizes membrane architecture to facilitate PUFA-driven signaling and multi-target metabolic modulation. This unique synergy highlights the therapeutic advantage of the complex lipid assembly in SBSO over single-component or highly saturated fatty acid oils in clinical applications.

#### 2.1.2. Palmitoleic Acid

Palmitoleic acid (POA, cis-9 C16:1, ω-7) is a characteristic monounsaturated fatty acid in SBPO, accounting for approximately 30–40% of the total fatty acids, although substantial variation has been reported among cultivars and geographical origins [9]. This concentration is markedly higher than that found in SBSO, which generally contains less than 5% POA. Advanced extraction methodologies, such as supercritical CO2 extraction, have been reported to better preserve thermolabile lipid components, including these valuable monounsaturated fatty acids, compared with conventional thermal processing methods [11,37].

Experimental studies have linked POA to a range of metabolic and dermatological biological activities. In cellular and animal models, POA has been reported to influence lipid metabolism and insulin-related pathways, particularly through PPARγ-associated signaling and the regulation of lipogenic gene expression [38]. In dermatological models, POA-containing preparations are shown to fortify epidermal barrier function, upregulate the expression of tight junction proteins, and attenuate the production of pro-inflammatory mediators, such as IL-6 and TNF-α [11]. Furthermore, macrophage studies suggest that POA facilitates cholesterol efflux by regulating the ABCA1 and ABCG1 transporters [39].

Human evidence regarding the therapeutic efficacy of POA remains limited and warrants cautious interpretation [10]. Epidemiological studies have highlighted associations between circulating POA levels and key metabolic health indicators, including insulin sensitivity and metabolic syndrome-related parameters [12]. Furthermore, several small-scale randomized controlled trials (RCTs) using purified POA supplementation have demonstrated improvements in inflammatory markers and serum lipid profiles [40,41]. Nevertheless, the robustness of these findings is constrained by relatively small sample sizes, short intervention periods, and heterogeneity in POA sources and formulations. Moreover, circulating POA levels are influenced not only by dietary intake but also by endogenous fatty acid desaturation, inherently complicating the interpretation of biomarker-based studies on POA.

Consequently, while preliminary evidence supports certain metabolic and dermatological effects of POA, these findings are predominantly derived from in vitro or animal models, with limited direct translatability to human physiology. Rigorously designed human intervention studies are thus urgently needed to delineate the specific contribution of SBPO-derived POA and to evaluate its long-term therapeutic relevance in clinical settings.

#### 2.1.3. Oleic Acid

Oleic acid (OA, cis-9 C18:1, ω-9) is a ubiquitous MUFA in both SBSO and SBPO, contributing to a total MUFA content of 20–40% [42,43]. Unlike the tissue-specific enrichment observed for palmitoleic or α-linolenic acids, OA serves as a foundational structural component that stabilizes the TAG matrices across both oil fractions. Analytical characterization has confirmed OA as the principal MUFA in TAG molecular species, predominantly occupying the sn-1 and sn-3 positions.

Genetic and geographic factors significantly influence OA concentrations by modulating adaptive desaturase expression profiles. For instance, Finnish subspecies typically exhibit 18–25% OA, whereas Mongolian populations—which have adapted to harsher climates by prioritizing PUFA synthesis for membrane fluidity—may contain as little as 2–8% [44]. This variability underscores a clear metabolic trade-off: colder environments favor PUFA biosynthesis, particularly α-linolenic acid, whereas temperate conditions promote higher MUFA accumulation.

Within the SBO lipid assembly, OA primarily functions as a structural scaffold rather than a primary bioactive driver. Its significance lies in a synergistic “dilution protection mechanism,” which is essential for the oxidative stability of SBSO. By intercalating within TAG molecules, OA effectively reduces the spatial density of polyunsaturated chains, thereby decreasing the efficiency of radical propagation and extending the oxidation induction period. This structural arrangement allows SBSO—despite containing 60–70% PUFAs—to demonstrate robust oxidative resistance compared to other high-PUFA oils, such as flaxseed oil [45]. Through this synergistic stabilization, oleic acid preserves the functional integrity of the labile active lipid components in SBO.

#### 2.1.4. Linoleic Acid

Linoleic acid (LA, C18:2, ω-6) is the predominant PUFA found in SBSO, with detailed regional variations in its content and other fatty acids presented in Table 2. It typically comprises 35–40% of the total fatty acids, although this proportion can range from 30–45% depending on cultivar and geographical origin. In contrast, it constitutes only 10–15% of SBPO [46]. Together with α-linolenic acid, these two essential PUFAs account for 60–70% of the total fatty acids in SBSO, making it one of the richest botanical sources of essential fatty acids. While near-infrared spectroscopy has been utilized for the rapid compositional screening of sea buckthorn fruit products [47], the precise quantification of LA in these extracted oils necessitates GC-FID or GC-MS analysis following transesterification.

In SBSO, LA functions primarily as a structural and metabolic precursor rather than a direct bioactive agent. As an essential fatty acid, LA fulfills several critical biological roles. Fundamentally, it reinforces membrane architecture by maintaining the fluidity of the epidermal lipid barrier. Metabolically, it serves as a precursor for arachidonic acid (AA) via Δ6-desaturase and elongase enzymatic pathways. However, its high degree of unsaturation creates a duality: while it underpins the nutritional value of SBSO, it simultaneously renders the oil highly susceptible to oxidation. Notably, the high essential fatty acid content of SBSO (60–70% PUFAs) is comparable to that of flaxseed oil, which has demonstrated cardiovascular benefits, including blood pressure attenuation and improved lipid profiles in patients with metabolic syndrome [48].

The antioxidant activity frequently associated with LA-containing oils is fundamentally a matrix effect. For instance, Akhter et al. [5] demonstrated that ethyl acetate-extracted SBSO exhibits substantial FRAP activity (up to 68%); however, this efficacy is primarily attributed to co-extracted phenolic compounds and tocopherols rather than LA itself. Chemically, LA is inherently pro-oxidative because of its highly reactive bis-allylic hydrogen atoms. Therefore, its stability depends heavily on the protective dilution provided by MUFAs and the synergistic antioxidants (vitamin E and polyphenols) found in the native SBSO matrix.

In dermatological applications, LA is essential for maintaining skin barrier integrity, primarily functioning as a ceramide-bound fatty acid within the stratum corneum intercellular lipid matrix. Its metabolite, 13-hydroxyoctadecadienoic acid (13-HODE)—generated via 15-lipoxygenase-mediated oxidation—modulates keratinocyte proliferation and differentiation through PPAR-α and TRPV1 receptor signaling pathways. This 13-HODE-specific pathway of LA represents a lipid mediator-driven approach that is complementary to polyphenol-based interventions [49].

In cardiovascular and metabolic investigations, SBO preparations rich in LA have demonstrated efficacy in modulating platelet aggregation, lipid metabolism, and oxidative stress parameters [52]. However, isolating the independent pharmacological contribution of LA from that of co-occurring α-linolenic acid, phytosterols, and tocopherols remains methodologically challenging. Consequently, while LA serves as a foundational nutritional component of SBSO, the overarching therapeutic properties of the oil are primarily driven by the synergistic action of its diverse lipid and non-lipid constituents. Moving forward, standardized compositional profiling and targeted mechanistic studies are essential to elucidate the specific role of LA within this complex multi-component system.

#### 2.1.5. Alpha-Linolenic Acid

Alpha-linolenic acid (ALA, C18:3, ω-3) is a prominent PUFA in SBSO and, together with LA, forms the dominant essential fatty acid fraction of the oil. Depending on cultivar, geographic origin, and extraction methodology, ALA generally accounts for 15–30% of the total fatty acids in SBSO, with certain genotypes exceeding 35% [50]. In stark contrast to SBPO, in which ALA is present only in trace amounts, SBSO is characterized by a pronounced ALA enrichment. This accumulation is driven by seed-specific desaturase activity during lipid biosynthesis.

Physiologically, ALA is an essential ω-3 polyunsaturated fatty acid that functions as the obligate metabolic precursor for longer-chain ω-3 derivatives, notably eicosapentaenoic acid (EPA) and docosahexaenoic acid (DHA). Although its endogenous hepatic conversion efficiency in humans is notoriously restricted [41], ALA exerts profound, independent biological activities. Beyond mere precursor functionality, it actively modulates membrane phospholipid architecture and attenuates pro-inflammatory signaling cascades. Through both direct and metabolite-mediated pathways, ALA orchestrates cardiometabolic regulation by optimizing lipid metabolism, enhancing endothelial function, and suppressing platelet aggregation [51]. Within dermatological contexts, ALA critically accelerates epidermal barrier restoration; it maintains necessary membrane fluidity, fortifies the intercellular lipid matrix of the stratum corneum, and precisely regulates local inflammatory responses to expedite tissue regeneration.

Despite its biological benefits, ALA is highly susceptible to lipid peroxidation due to its three cis double bonds, making it one of the most oxidation-sensitive fatty acids within SBSO. Therefore, its oxidative stability depends heavily on the native antioxidant matrix of the oil—comprising tocopherols, carotenoids, and phytosterols—as well as the structural dilution effect provided by monounsaturated fatty acids such as oleic acid [53]. Consequently, ALA acts as a critical determinant of both the functional value and oxidative vulnerability of SBSO, underscoring the importance of optimizing processing and storage conditions to preserve its bioactivity and minimize autoxidative degradation.

### 2.2. Vitamins

Sea buckthorn is widely acclaimed as a “natural vitamin treasury,” a designation underpinned by the strategic distribution of vitamins across its anatomical components: SBSO, SBPO, and juice/pomace (Table 3). Notably, while the aqueous fraction of sea buckthorn is characterized by a prolific vitamin C content—with ascorbic acid levels reaching unprecedented concentrations of 400–2500 mg/100 g [54]—this hydrophilic antioxidant is excluded from the lipidic phase. Consequently, less than 10 mg/kg of ascorbic acid is found in the oils, reflecting its selective retention in the aqueous phase during industrial processing [55]. Conversely, the extracted oils serve as potent reservoirs for lipophilic vitamins, manifesting a complementary phytochemical heterogeneity that maximizes the plant’s overall nutritional density.

Vitamin E, comprising tocopherols and tocotrienols, was the main vitamin present in both oil fractions. SBSO contains 100–300 mg/kg tocopherols, with γ-tocopherol accounting for 60–80% of the mixture, followed by α- and δ-forms [56]. In contrast, SBPO has lower but still significant levels (50–150 mg/kg) and features a more balanced α/γ ratio. These concentrations are comparable to or higher than those reported for many common vegetable oils, including olive oil, which contains 50–200 mg/kg. As lipid-soluble antioxidants, tocopherols protect PUFAs from peroxidation by donating hydrogen to lipid radicals. γ-Tocopherol demonstrates superior anti-inflammatory activity, particularly in COX-2 suppression, compared to α-tocopherol.

The vitamin A activity in SBPO is primarily derived from carotenoids, especially β-carotene, zeaxanthin, and lycopene, with concentrations reaching 100–500 mg/kg, among the highest found in plant oils. In contrast, SBSO contains significantly lower levels of carotenoids (1–5 mg/kg) due to the limited chromoplast content of the seeds. These provitamin A compounds not only give the oil its distinctive orange-red hue, but also offer photoprotection, immune modulation, and vision support [18].

Bioflavonoids, historically referred to as “vitamin P,” are predominantly sequestered in sea buckthorn juice, pomace, and other polar fractions rather than in refined oils. The primary compounds include isorhamnetin, quercetin, and kaempferol derivatives [19]. Due to their hydrophilic nature, these compounds exhibit low solubility in purified oil fractions; however, trace amounts may be retained in minimally processed products, such as crude oils.

Water-soluble vitamins, notably vitamin C and the B-complex group, are significantly more abundant in whole berry products and processing residues than in refined oils [20]. Consequently, the nutritional profile of sea buckthorn-derived products is heavily modulated by the extraction methodology and the degree of refinement. While cold-pressed oils selectively retain lipid-soluble vitamins, whole-fruit preparations and byproduct-derived extracts offer a broader spectrum of essential micronutrients.

### 2.3. Carotenoids

Carotenoids represent the defining bioactive constituents of SBPO, distinguishing it from both SBSO and most conventional vegetable oils. SBPO exhibits a total carotenoid content of 100–500 mg/kg—ranking it among the most concentrated botanical sources—whereas SBSO contains negligible amounts (<5 mg/kg) due to the lack of chromoplasts within the seed matrix [21]. This tissue-specific enrichment establishes SBPO as a potent natural colorant and nutraceutical matrix, with its potency varying significantly according to cultivar, fruit maturity, and environmental factors, such as altitude, temperature, and solar radiation.

The carotenoid composition of SBPO is notably diverse, encompassing β-carotene (provitamin A, 20–40%), zeaxanthin (macular pigment, 15–25%), lycopene (5–15%), β-cryptoxanthin (10–20%), lutein (10–20%), and minor α-/γ-carotene isomers [57]. In contrast to oils dominated by a single carotenoid, such as palm oil, which is primarily β-carotene, SBPO offers a comprehensive carotenoid matrix that exhibits synergistic biological activities. Within this complex matrix, provitamin A is predominantly facilitated by β-carotene and β-cryptoxanthin, which are converted to retinal through central cleavage by β-carotene 15,15′-monooxygenase (BCMO1). The substantial bioaccessibility of β-carotene in SBPO, estimated at 30–50% micellarization, contributes to vision, immune function, and epithelial integrity. However, the efficiency of direct conversion is influenced by the dietary lipid matrix and genetic variants [58].

In addition to their provitamin A activity, carotenoids in SBPO exert direct biological effects, including antioxidant protection through singlet oxygen quenching and radical scavenging, with zeaxanthin demonstrating superior membrane-stabilizing properties. They also modulate anti-inflammatory responses via the regulation of the NF-κB and Nrf2 pathways, provide skin photoprotection by filtering blue light and mitigating UV-induced oxidative damage, and regulate metabolism through the modulation of PPARγ and SREBP-1c, thereby influencing adipocyte differentiation and hepatic lipid metabolism [59]. These biological activities are further enhanced by tocopherols and PUFAs in SBPO, which stabilize carotenoids and improve their bioavailability. Tocopherols protect carotenoids from oxidative degradation during storage and digestion. Furthermore, SBPO confers cardiovascular protection through dual carotenoid mechanisms: LDL oxidation inhibition (zeaxanthin > β-carotene) and ABCA1-mediated cholesterol efflux enhancement.

In cosmetics, the carotenoid content of SBPO imparts a characteristic orange-red hue and offers intrinsic photoprotective properties through UV absorption. Furthermore, it exerts anti-aging benefits by stimulating collagen synthesis. These attributes distinguish SBPO from SBSO, broadening its application in sunscreens, serums, and regenerative cream formulations. However, the stability of carotenoids is constrained by their susceptibility to photo-oxidation and thermal degradation, necessitating strategies such as dark packaging, nitrogen flushing, and cold-pressed extraction to maintain their bioactivity.

Notably, the native triacylglycerol (TAG) matrix of SBPO facilitates the formation of mixed micelles during digestion, significantly enhancing carotenoid bioaccessibility. While classic human clinical studies on similar natural lipid matrices reveal a profound increase in carotenoid absorption—often two- to three-fold—compared to low-fat or aqueous fruit preparations [60], robust human pharmacokinetic data directly comparing the absorption kinetics of SBPO with synthetic β-carotene formulations are currently limited [61]. Overall, while SBPO represents a promising natural source of dietary carotenoids, further standardized human intervention trials are warranted to clarify the bioavailability, metabolic fate, and long-term physiological relevance of its specific carotenoid fractions.

### 2.4. Polyphenolic Compounds

A critical distinction must be made regarding the polyphenolic compounds in sea buckthorn: while they are celebrated for their potent antioxidant activity, their hydrophilic nature ensures they remain virtually absent from refined SBO (<5–10 mg/100 g). The key implication is that polyphenols are not inherently lipophilic bioactives; consequently, fully harnessing their synergistic health benefits alongside SBO necessitates deliberate formulation strategies—such as oil-in-water emulsions, pomace-fortified oils, or co-extracts—which leverage the plant’s high-value by-products.

These by-products—comprising seed meal, pomace, and juice—represent the primary reservoirs of sea buckthorn polyphenols. The total polyphenol content (TPC) reaches 93–200 mg/100 g in seed meal and 100–500 mg/100 g in pomace, underscoring their potential as robust functional ingredients [62]. These compounds predominantly consist of flavonol glycosides—notably isorhamnetin-3-O-rutinoside, quercetin-3-O-glucoside, and kaempferol derivatives—hydroxycinnamic acids (ferulic, caffeic, and p-coumaric acids), and condensed tannins (procyanidins), with their distribution exhibiting significant variations based on tissue type, cultivar, and geographical origin [63].

Consequently, the term “polyphenol content of SBO” is often misleading. Due to their inherently low lipid solubility, most polyphenols are partitioned out during extraction, as refined oils lack the polar environment necessary for their retention. Nevertheless, cold-pressed oils and unrefined mixtures may retain trace phenolics—likely stabilized by phospholipids (5–15 mg/100 g)—which minimally contribute to oxidative stability through mechanisms such as metal chelation and radical scavenging at the oil–water interface. Ultimately, the intrinsic antioxidant activity of SBO is predominantly driven by tocopherols and carotenoids, with polyphenols exerting a synergistic effect only in whole berry extracts or fortified oil–pomace formulations.

Polyphenol-rich extracts derived from sea buckthorn pomace and seed meal demonstrated significant bioactivity, independent of the oil matrix. These extracts exhibit: (1) antioxidant capacity, with ORAC values ranging from 200 to 400 μmol TE/g and DPPH IC_50_ values between 10 and 50 μg/mL, which strongly correlate with total phenolic content [22]; (2) anti-inflammatory effects through the suppression of NF-κB and MAPK pathways in macrophage models; (3) modulation of gut microbiota, where flavonol glycosides enhance the growth of *Bifidobacterium* and *Lactobacillus* while inhibiting pathogenic species [23]; and (4) inhibition of metabolic enzymes, including α-amylase, α-glucosidase, and ACE, thereby supporting anti-diabetic and anti-hypertensive applications [64].

When successfully extracted from pomace and seed meal, these polyphenol-rich fractions demonstrate profound bioactivity, independent of the oil matrix. These extracts exhibit (1) antioxidant capacity, with ORAC values ranging from 200 to 400 μmol TE/g and DPPH IC_50_ values between 10 and 50 μg/mL, which strongly correlates with total phenolic content [65]; (2) anti-inflammatory effects through the suppression of NF-κB and MAPK pathways in macrophage models; (3) modulation of gut microbiota, where flavonol glycosides enhance the growth of *Bifidobacterium* and *Lactobacillus* while inhibiting pathogenic species [66]; and (4) inhibition of metabolic enzymes, including α-amylase, α-glucosidase, and ACE, thereby supporting anti-diabetic and anti-hypertensive applications [59].

Processing plays a pivotal role in bridging the solubility gap between hydrophilic bioactives and lipid formulations. Key methodologies include microbial fermentation, which enhances the bioavailability of free phenolic acids through enzymatic glycoside hydrolysis; thermal treatment, which induces complex Maillard–polyphenol conjugation with divergent bioactive outcomes; and phospholipid complexation, which augments the intestinal permeability of isorhamnetin and quercetin by 3- to 5-fold [67]. These advanced delivery systems constitute engineered nutraceuticals that successfully integrate the distinct functional profiles of sea buckthorn’s lipid and aqueous fractions.

### 2.5. Sterol Compounds

Phytosterols are distinctive bioactive components of SBSO, with total concentrations ranging from 10,000 to 15,000 mg/kg (1–1.5%), among the highest observed in vegetable oils. In contrast, SBPO contains significantly lower levels of these compounds, between 1000 and 3000 mg/kg [68]. The predominant sterols include β-sitosterol (60–70%), campesterol (15–25%), stigmasterol (5–10%), and Δ5-avenasterol (5–10%), with minor contributions from brassicasterol and sitostanol. This compositional profile is similar to that of rice bran and wheat germ oils, establishing SBSO as a functional source of phytosterols. The extraction methodology significantly influenced sterol recovery. Solvent extraction using hexane and supercritical CO_2_ extraction typically yields higher sterol concentrations (12,000–14,000 mg/kg). In contrast, cold pressing produced sterol levels approximately 10–20% lower because of incomplete cellular disruption. Saponification, a necessary step for the analysis of free sterols, hydrolyzes steryl esters, revealing that 50–70% of SBSO sterols are present in esterified forms, specifically as fatty acyl steryl esters [13]. These esterified forms enhance lipid solubility and oxidative stability compared to free sterols.

The cholesterol-lowering mechanism of SBSO sterols is primarily driven by the competitive inhibition of intestinal cholesterol absorption. Specifically, β-sitosterol and campesterol—owing to their structural homology to cholesterol—displace cholesterol from mixed micelles, thereby attenuating its incorporation into chylomicrons. According to the clinical consensus guidelines from the European Atherosclerosis Society (EAS) and comprehensive meta-analyses, a daily intake of 2–3 g of phytosterols reliably reduces low-density lipoprotein cholesterol (LDL-C) levels by 10–12% [14]. While this therapeutic dosage is theoretically achievable through the consumption of 20–30 g of SBSO, such high intake imposes substantial caloric burdens (approximately 180–270 kcal/day), potentially complicating standard dietary management. Therefore, SBSO is more appropriately utilized as a specialized functional lipid ingredient—integrated into emulsions, functional foods, or supplements—to contribute to the total requisite daily intake of dietary sterols [15].

Beyond lipid management, other purported therapeutic properties of SBSO sterols remain confined to preclinical exploration. The metabolic effects of SBSO sterols and their derivatives, including stanols and steryl ferulates, extend to glycemic regulation by enhancing insulin sensitivity in adipocyte and hepatocyte models. This improvement is potentially mediated through the activation of PPARγ and suppression of inflammatory cytokines, such as IL-6 and TNF-α [60,69]. The intestinal absorption of phytosterols in humans is notoriously poor (typically less than 5%), meaning that achieving the high systemic concentrations (50–200 μM) required for in vitro apoptotic or intense anti-inflammatory activity is physiologically improbable through dietary SBO intake alone [70]. Therefore, while isolated sterols exhibit promising bioactivity, therapeutic claims extending beyond localized gastrointestinal effects (such as cholesterol blocking) require rigorous clinical validation.

Furthermore, assumptions regarding the synergistic interactions of these lipophilic bioactives must account for their spatial distribution within the plant matrix. Notably, claims of direct synergy between sterols and squalene within a single native oil fraction are biochemically unsubstantiated due to their reciprocal distribution. These compounds are highly compartmentalized: SBSO is sterol-rich but squalene-poor, whereas SBPO is squalene-rich but sterol-poor [71]. Therefore, any observed synergistic antioxidant effects generally reflect the use of whole-berry extracts or intentional oil blends rather than unmodified single-oil compositions. This tissue-specific compartmentalization underscores the necessity of strategic oil blending or residue recombination to maximize the phytochemical diversity and functional efficacy of SBO-derived products.

## 3. The Therapeutic Application of SBO

### 3.1. Antioxidant and Anti-Inflammatory Effects

**Figure 3:**
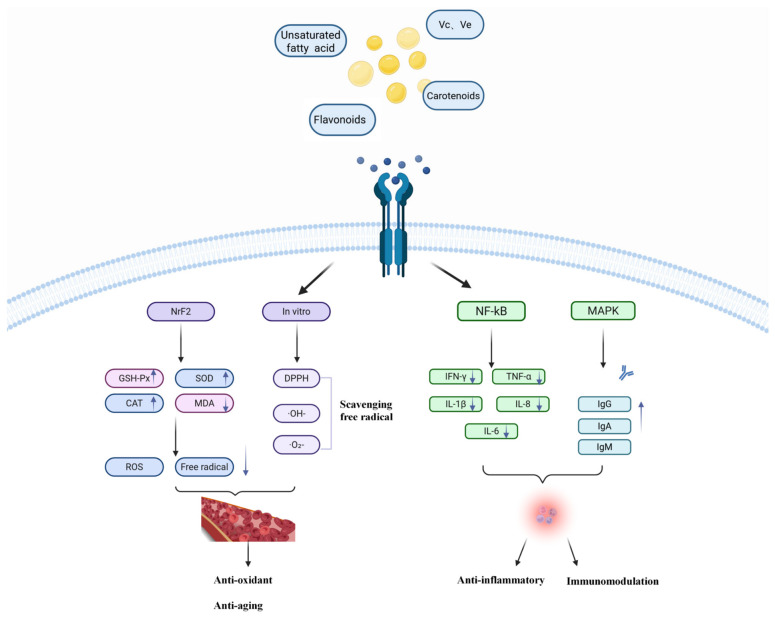
Mechanism of anti-inflammatory and antioxidant action of SBO.

The antioxidant and anti-inflammatory activities of sea buckthorn products arise from the distinct bioactive profiles present in different fractions of the plant [72]. Comprehensive reviews suggest that sea buckthorn possesses therapeutic potential owing to the synergistic interactions between oil-soluble and polar constituents. However, to accurately interpret these bioactivities, a clear distinction must be made between the lipid-mediated effects of refined oils and the polar-mediated effects of the crude extracts. These mechanisms include the neutralization of reactive oxygen species (ROS), inhibition of lipid peroxidation, and modulation of inflammatory pathways, as summarized in Figure 3.

When examining lipid-mediated mechanisms within the refined fractions (SBSO and SBPO), the antioxidant and anti-inflammatory activities operated exclusively within the lipid matrix. In SBSO, γ-tocopherol (100–300 mg/kg) protects abundant PUFAs (60–70% of total fatty acids) from peroxidation by donating hydrogen atoms to lipid peroxyl radicals, forming stable tocopheroxyl radicals that interrupt chain reactions. This mechanism is complemented by α-tocopherol recycling and the sacrificial role of PUFAs as oxidative buffers [16,73]. In SBPO, singlet oxygen quenching is predominantly mediated by carotenoids: β-carotene and zeaxanthin absorb ^1^O_2_ energy and dissipate it as heat without undergoing chemical transformation. Regarding inflammation, the oil fractions act primarily through lipid signaling and transcriptional modulation: ALA in SBSO acts as a precursor for specialized pro-resolving mediators (resolvins, protectins, and maresins), whereas β-carotene in SBPO modulates anti-inflammatory pathways.

Diverging from the lipid-based pathways, the pharmacological landscape of crude extracts and processing co-products is governed by potent polar-mediated mechanisms. Extracts sourced from the whole berry, seed meal, and pomace constitute a dense phytochemical matrix of flavonol glycosides, phenolic acids, and polymeric tannins. Mechanistically, these polar constituents exert dual inhibition on the cyclooxygenase (COX-1/2) and lipoxygenase (5-LOX) pathways, effectively blunt the production of pro-inflammatory cytokines, and attenuate the subsequent inflammatory cascade [17].

This fundamental compositional divergence creates a critical translational gap between oils and extracts in vivo. The key distinction lies in the absolute absence of the flavonoid-rich polar fraction in the refined oils (SBSO and SBPO). Many animal models demonstrating profound systemic effects, such as reduced serum MDA, suppression of hepatic TNF-α, and significant alleviation of colitis, utilize crude extracts, oil–residue mixtures, or fermented products, wherein oil-soluble and polyphenolic components interact synergistically [74]. Isolated refined oils exhibit diminished systemic anti-inflammatory effects compared to mixed matrices.

This compositional distinction is paramount when interpreting in vivo results. Numerous animal studies reporting reductions in oxidative stress markers or inflammatory mediators have employed crude extracts, fermented products, or complex preparations rather than purified oil fractions [75]. Consequently, the biological outcomes observed in these models likely reflect the synergistic contributions of multiple chemical classes. Currently, clinical evidence specifically attributing systemic antioxidant and anti-inflammatory effects to refined SBO remains scant, underscoring the necessity for further rigorous, controlled human trials.

### 3.2. Cardiovascular Protective Effect

The interpretation of the cardiovascular effects reported for sea buckthorn products is complicated by substantial differences in the composition of refined oils, whole-fruit extracts, and polyphenol-rich fractions. Therefore, distinguishing between the lipid-associated effects of SBSO and SBPO and the vascular activities reported for flavonoid-rich polar extracts is crucial.

Within the lipid-centric mechanisms of SBSO, the cardiovascular benefits of refined SBSO are fundamentally driven by its high PUFA and phytosterol content. SBSO comprises 60–70% PUFAs, including ALA and LA. ALA serves as a precursor for EPA and DHA synthesis, leading to the production of specialized pro-resolving lipid mediators that mitigate vascular inflammation by reducing tumor necrosis factor-alpha (TNF-α and IL-6) secretion. Furthermore, these PUFAs enhance lipoprotein kinetics by upregulating the hepatic LDL receptor expression. In addition, the abundant phytosterols in SBSO competitively inhibit intestinal cholesterol absorption by displacing cholesterol from mixed micelles [76]. Additionally, gamma-tocopherol in SBSO helps preserve the baseline nitric oxide bioavailability by scavenging peroxynitrite and inhibiting COX-2.

In contrast to PUFA-dominant SBSO, SBPO confers cardiovascular protection through a completely different yet complementary lipid matrix. SBPO is highly enriched in MUFAs, particularly palmitoleic acid (ω-7, 30–40%), which improves systemic lipid profiles, attenuates hepatic steatosis, and enhances insulin sensitivity. Furthermore, unlike SBSO, SBPO contains extremely high levels of lipophilic carotenoids (e.g., zeaxanthin and β-carotene) [77]. These carotenoids are incorporated into circulating lipoproteins, effectively protecting low-density lipoproteins (LDL) from oxidative modification, a critical initiating step in atherosclerosis. Thus, while SBSO primarily acts as a cholesterol blocker and PUFA donor, SBPO functions as an antioxidant protector and MUFA metabolic regulator.

Moving beyond the lipid-centric effects of refined oils, potent vascular modulatory actions are exclusively attributable to the polar fractions. Mechanisms such as direct endothelial NO release, active vasodilation, and angiotensin-converting enzyme (ACE) inhibition are frequently—and erroneously—ascribed to SBO [78,79]. These actions are pharmacologically governed by hydrophilic flavonoids (notably quercetin) and phenolic acids. Quercetin directly stimulates endothelial NO synthesis and activates the guanylate cyclase-cGMP signaling cascade to induce vascular smooth muscle relaxation [80]. Similarly, ACE inhibition is typically mediated by specific peptides and phenolic-rich fractions. Because refined SBSO and SBPO contain only trace flavonoid concentrations (<5–10 mg/kg), they fundamentally lack the biochemical apparatus required to trigger these direct antihypertensive pathways.

This distinction is paramount when interpreting animal and clinical data derived from whole-berry preparations, fermented products, or hybrid extracts, in which multiple bioactive classes co-exist [81,82]. Consequently, the cardiovascular outcomes reported for sea buckthorn products likely arise from a concerted interplay between lipid-soluble and polar constituents. Further well-designed human studies are warranted to disentangle the relative contributions of refined oils versus polyphenol-rich extracts.

### 3.3. Effects on the Digestive System

Sea buckthorn products provide comprehensive gastrointestinal (GI) protection through intricate interactions between lipid-soluble and polar bioactive compounds. To navigate this dense biochemical matrix, it is essential to delineate the therapeutic applications of refined oils versus whole-matrix preparations. This can be achieved by categorizing their effects into three distinct functional pathways: lipid-mediated (primarily focused on mucosal integrity), carotenoid-mediated (centered on oxidative defense), and polyphenol-mediated (focused on inflammatory modulation and microbiome health).

#### 3.3.1. Lipid-Mediated Effects (SBSO)

The structural gastrointestinal benefits of refined oils are largely associated with their high phytosterol content (10,000–15,000 mg/kg) found in SBSO. Phytosterols, including β-sitosterol, campesterol, and stigmasterol, are structural analogs of cholesterol. They actively integrate into the lipid bilayers of enterocyte membranes, modulating membrane fluidity and enhancing tight junction integrity [83]. This enhancement of the barrier function significantly reduces mucosal permeability to gastric acid, pepsin, and bacterial endotoxins. in vitro studies have further indicated that β-sitosterol improves epithelial cell viability under acidic stress conditions [84]. Additionally, the unsaturated fatty acids present in both SBSO and SBPO, particularly oleic acid (15–20%), contribute to mucosal protection by inhibiting the activation of nuclear factor kappa B and suppressing the production of pro-inflammatory cytokines in intestinal epithelial cells [85].

#### 3.3.2. Carotenoid-Mediated Effects (SBPO)

In contrast to sterol-driven SBPO, SBPO contributes to gastrointestinal protection primarily through its dense carotenoid content (100–500 mg/kg). Specifically, β-carotene and β-cryptoxanthin are potent provitamin A precursors. Upon cellular uptake, they support intestinal epithelial proliferation and differentiation via retinoic acid receptor (RAR) signaling, which actively accelerates mucosal regeneration following gastric injury [86]. While broader clinical and nutritional studies indicate that carotenoid-rich oils generally enhance ulcer healing kinetics, controlled trials investigating the specific ulcer-healing endpoints of isolated SBPO remain essential for future research [87].

#### 3.3.3. Polyphenol and Polar-Mediated Effects (Extracts and Juice)

A critical distinction must be made regarding the mucus-stimulating, acid-secretory, and prebiotic effects that are traditionally attributed to SBO. These benefits are primarily derived from the polar fractions (juice, pomace, and seed meal) rather than from refined lipids. Specifically, anti-inflammatory flavonoids, such as isorhamnetin and quercetin glycosides, stimulate protective mucus secretion and modulate inflammatory signaling pathways [88]. Furthermore, organic acids in juice stimulate normal gastric acid secretion, while complex polysaccharides function as prebiotics, actively promoting the growth of beneficial gut microbiota, such as *Bifidobacterium* [89].

#### 3.3.4. Formulation Strategies for Gastrointestinal Delivery

From a delivery science perspective, isolated refined oils may exhibit limited mucosal adhesion and stability within the harsh gastric milieu. The bioaccessibility of lipophilic bioactives—notably sterols, carotenoids, and tocopherols—is critically contingent upon food-grade colloidal systems. Formulations such as oil-in-water (O/W) emulsions, microencapsulation, and liposomal systems shield these bioactives from premature enzymatic degradation and facilitate prolonged interactions with mucosal surfaces [90,91]. Consequently, integrated delivery strategies, including oil-in-juice emulsions and phospholipid-complexed formulations, have emerged as pivotal paradigms. These multi-matrix approaches synergistically combine the membrane-stabilizing sterols of SBSO, the regenerative carotenoids of SBPO, and the mucosal-protective polyphenols of polar extracts, yielding significantly enhanced gastrointestinal benefits compared to stand-alone refined oils.

### 3.4. Other Potential Therapeutic Applications

#### 3.4.1. Immunomodulation

The immune system maintains physiological homeostasis through highly coordinated cellular and humoral responses [92]. The immunomodulatory and anti-inflammatory properties of sea buckthorn are synergistically mediated by both its polar extracts and lipophilic fractions. Regarding the polar constituents, sea buckthorn polysaccharides have been shown to potentiate T-lymphocyte proliferation and upregulate the secretion of key cytokines, signaling active cellular immune stimulation [93]. Furthermore, sea buckthorn-derived polyphenols and polysaccharides collectively modulate macrophage polarization and attenuate pro-inflammatory signaling pathways [94].

In addition to these polar extracts, SBO exhibits notable immunomodulatory effects. in vitro, the administration of SBO at non-cytotoxic concentrations (<25 μL/mL) in LPS-stimulated THP-1 cells effectively suppressed the release of pro-inflammatory cytokines [95]. These cellular mechanisms translate well to the in vivo environment. For instance, in animal models of localized inflammation, combined oral (100–200 mg/kg) and topical (20 μL) administration of SBO significantly reduced localized inflammatory lesions and mitigated systemic cytokine-related responses [96].

Despite these promising preclinical findings, current evidence relies predominantly on in vitro assays and animal-model studies. However, robust human clinical evidence remains scarce. Therefore, definitive conclusions regarding the immunomodulatory therapeutic efficacy of sea buckthorn fractions require rigorous clinical validation in future studies.

#### 3.4.2. Preclinical Observations in Oncology

While oxidative stress and chronic inflammation facilitate tumorigenesis by inducing DNA damage and sustaining a pro-carcinogenic microenvironment [97], claims regarding the oncological applications of sea buckthorn must be strictly grounded in specific preclinical evidence rather than broad generalizations. Currently, the evidence base is confined to in vitro and murine models. In these controlled settings, polar constituents have demonstrated targeted antiproliferative activities. For instance, in vitro studies on human hepatocellular carcinoma (HepG2) cells have shown that total sea buckthorn flavonoids (rich in isorhamnetin and quercetin) significantly reduce cancer cell viability and induce caspase-dependent apoptosis by suppressing the PI3K/Akt and JAK pathways [98]. Similarly, specific sea buckthorn polysaccharides modulated the tumor microenvironment in murine models by enhancing macrophage phagocytosis and immune surveillance [99].

Conversely, refined oils (SBSO and SBPO) lack direct cytotoxic activity against malignant cells. They contribute indirectly to these preclinical models; their lipophilic antioxidant matrices (tocopherols and carotenoids) provide baseline cellular protection by reducing oxidative DNA damage [100]. Crucially, extrapolating these localized in vitro responses to systemic human cancer prevention is highly speculative and requires further investigation. Because achieving the high micromolar concentrations required for in vitro apoptosis is physiologically improbable through dietary ingestion alone, sea buckthorn products must be rigorously classified as complementary dietary antioxidants, rather than therapeutic oncological agents.

#### 3.4.3. Skin Protection and Repair

The barrier function of the stratum corneum is critically reliant on intercellular lipids such as ceramides, cholesterol, and free fatty acids. The essential fatty acids in SBSO and the rare palmitoleic acid in SBPO provide vital structural components for the skin. Specifically, linoleic acid integrates into ceramide 1-linoleate, thereby maintaining lamellar organization and mechanically reducing transepidermal water loss (TEWL) [100].

To substantiate this mechanistic rationale with the latest objective evidence, recent human clinical trials and pharmacological evaluations have firmly established the dermatological efficacy of the SBO. A 2024 randomized controlled trial (RCT) demonstrated that daily oral supplementation with SBO significantly improved objective skin parameters, yielding quantifiable enhancements in stratum corneum hydration and skin elasticity compared with the baseline [16]. Furthermore, a comprehensive pharmacological evaluation in 2024 confirmed that the distinct lipid matrix of SBO operates beyond simple physical barrier repair; it actively modulates localized inflammatory pathways, significantly reducing erythema and accelerating tissue regeneration in inflammatory skin conditions such as atopic dermatitis [101]. Notably, these macroscopic improvements in barrier recovery are often maximized when comprehensive formulations that retain residual polar bioactives alongside the lipid matrix are used [49].

#### 3.4.4. UV Protection and Photostability

Photooxidative skin damage is biochemically characterized by the generation of reactive oxygen species (ROS), extensive lipid peroxidation, and subsequent activation of matrix metalloproteinases [92]. The native lipid fractions of sea buckthorn exhibit specific photoprotective properties, driven by their distinct antioxidant profiles. In SBPO, the carotenoid fraction, primarily comprising β-carotene, zeaxanthin, and lycopene, functions as an optical filter to absorb UV radiation and directly quenches singlet oxygen. Concurrently, the high concentration of tocopherols in SBSO terminates lipid peroxidation chain reactions by scavenging peroxyl radicals [102].

These biochemical mechanisms were substantiated by controlled pharmacological evaluations. in vitro assays using UV-irradiated human skin fibroblasts and keratinocytes demonstrated that SBO administration significantly attenuated intracellular ROS accumulation and preserved cellular membrane lipid integrity, thereby preventing UV-induced redox imbalances [103]. Furthermore, in vivo preclinical murine models of UV-induced photoaging quantitatively confirmed these findings. Topical application of the oil yielded statistically significant reductions in cutaneous malondialdehyde (MDA), the primary end product of lipid peroxidation. This reduction is accompanied by the restoration of endogenous antioxidant enzyme activities, specifically the upregulation of superoxide dismutase (SOD) and glutathione peroxidase (GSH-Px) [104]. Collectively, these objective biochemical parameters provide a robust pharmacological rationale for the photoprotective capacity of sea buckthorn oil against UV-induced cutaneous damage.

## 4. The Utilization of By-Products of SBO

### 4.1. Overview of By-Products

**Figure 4:**
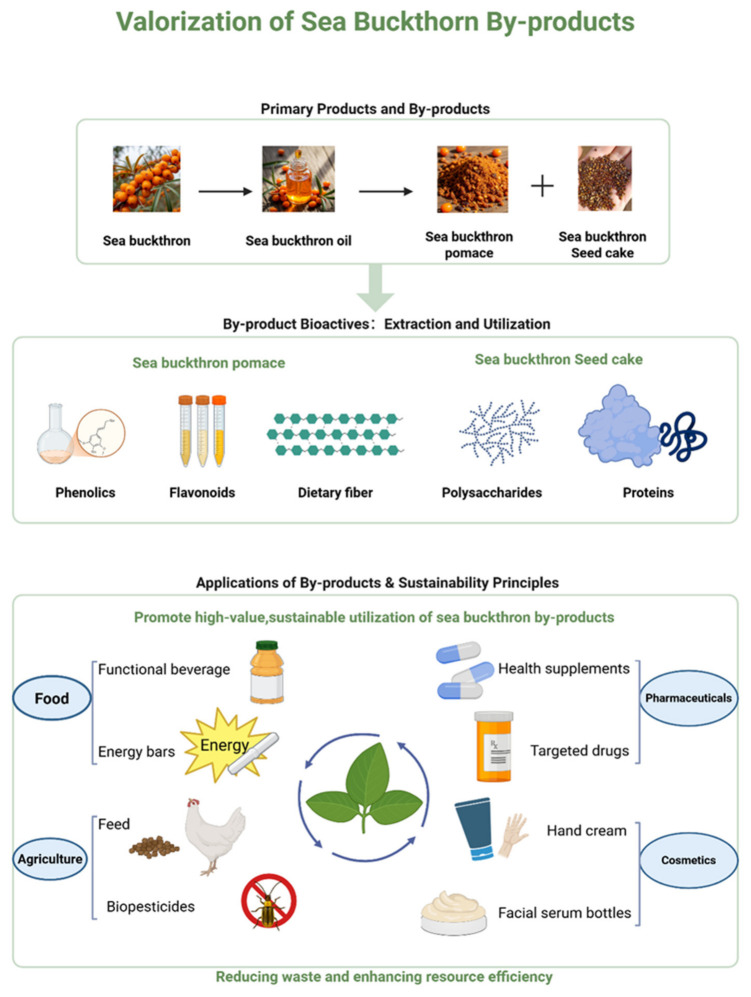
Main components and functions of sea buckthorn pomace and seed meal.

SBO extraction generates substantial quantities of pomace (the fibrous residue remaining after pulp oil extraction) and seed meal (the protein-rich residue after SBSO extraction) [105,106]. Traditionally dismissed as low-value waste, these by-products retain significant amounts of bioactive compounds—including polyphenols, dietary fibers, proteins, and residual lipids—which underpin their functional applications in foods, nutraceuticals, and animal nutrition [107,108]. The valorization of these residues aligns with circular economy principles, offering strategic opportunities to mitigate environmental impact and enhance resource efficiency (Figure 4).

Pomace constitutes approximately 20–30% (wet basis) of the processed fruit weight and consists of fruit skins, residual pulp, and occasionally unseparated seeds. It is rich in dietary fiber (40–70% dry weight) and is primarily composed of pectic polysaccharides, cellulose, and hemicellulose [24]. The fiber fraction can support prebiotic activity and improve the textural properties of food formulations. Pomace also retains residual polyphenols and carotenoids, which may contribute to its antioxidant properties when incorporated into food or feed applications [109,110].

Seed meal, derived from defatted seed kernels and seed coats, is protein-rich (40–70% crude protein, on a dry weight basis) and contains a well-balanced amino acid profile, including essential amino acids such as lysine and leucine [27]. Enzymatic hydrolysis of these proteins produces bioactive peptides with reported metabolic regulatory activity, including α-glucosidase and dipeptidyl peptidase-IV (DPP-IV) inhibitory effects, suggesting their potential utility in glycemic control and diabetes-targeted nutraceuticals [28]. Residual lipids in seed meal (10–15%) are enriched with unsaturated fatty acids and contain tocopherols and essential minerals (K, Ca, Mg), which may improve oxidative stability and nutritional density [29,30,31].

Ultimately, the complementary characteristics of these by-products—pomace being rich in fiber and antioxidants and seed meal being abundant in protein and PUFAs—facilitate integrated valorization strategies [111]. These approaches include combined fermentation processes to produce protein–fiber concentrates, enzymatic hydrolysis for bioactive peptides and phenolic extracts, and their direct incorporation into composite foods to optimize resource efficiency and economic benefits.

### 4.2. Extraction and Application of Bioactive Components in By-Products

The efficient recovery of bioactive compounds from sea buckthorn by-products is essential for enhancing both resource utilization and economic value. Following juice or oil extraction, sea buckthorn pomace and seed meal remain important reservoirs of phenolic acids, flavonoids, flavonol glycosides, proanthocyanidins, carotenoids, dietary fibers, proteins, bioactive peptides, tocopherols, and residual lipids [112,113,114]. Commonly identified phenolic acids include gallic acid, protocatechuic acid, caffeic acid, and ferulic acid, whereas isorhamnetin and its glycosides are recognized as characteristic marker compounds of sea buckthorn-derived materials. Seed meal is particularly enriched in proanthocyanidins, which exhibit notable antioxidant activity and inhibitory effects against carbohydrate-hydrolyzing enzymes [115,116]. Reported total phenolic contents in sea buckthorn pomace extracts range from approximately 100 to 500 mg/100 g. Under optimized extraction conditions, flavonoid and flavonol contents may reach 325.08 μg/g and 217.55 μg/g, respectively, while antioxidant capacities have been reported with ORAC values of 200–400 μmol TE/g and DPPH IC_50_ values ranging from 10 to 50 μg/mL [117].

Extraction methodology strongly influences both the recovery yield and functional properties of bioactive compounds derived from sea buckthorn by-products. Conventional solvent extraction using aqueous ethanol, methanol, or glycerol remains widely applied for the recovery of polar phenolics. Nevertheless, environmentally friendly extraction strategies have attracted increasing attention in recent years owing to their improved efficiency and reduced solvent burden [118,119]. Supercritical CO_2_ extraction (SFE-CO_2_) is particularly effective for recovering lipophilic constituents, including residual tocopherols, carotenoids, phytosterols, and unsaturated fatty acids, while minimizing solvent residues and thermal degradation of sensitive compounds [25]. For the extraction of polar phytochemicals, pressurized liquid extraction with ethanol (PLE-EtOH) has been reported to achieve approximately 57% phenolic recovery from seed fractions under optimized conditions while maintaining the stability of glycosidic structures [26,120]. In addition, enzyme-assisted extraction (EAE), commonly employing cellulase and pectinase, further enhances the release of water-soluble polysaccharides and bound phenolic compounds from complex plant matrices.

Beyond extraction efficiency, improving the stability and bioaccessibility of recovered bioactives has also become an important research focus. Sea buckthorn pomace represents an important source of carotenoids, particularly zeaxanthin esters, β-carotene, γ-carotene, and lycopene. Encapsulation using alginate hydrogel systems has been shown to significantly improve carotenoid stability and bioaccessibility, with total carotenoid bioaccessibility ranging from 29.5% to 42.1%. Among the identified compounds, zeaxanthin dipalmitate and β-carotene exhibited particularly high bioaccessibility values of 66.05% and 51.42%, respectively [121]. These findings support the potential application of pomace-derived carotenoids in functional foods and nutraceutical delivery systems.

Dietary fiber and protein-rich fractions recovered from sea buckthorn by-products also exhibit considerable functional potential. Dietary fiber constitutes a major component of SBP and is composed primarily of soluble pectic polysaccharides (10–30%) and insoluble cellulose and hemicellulose fractions (70–90%) [122]. The soluble dietary fiber fraction can function as a prebiotic substrate, promoting short-chain fatty acid (SCFA) production during colonic fermentation and contributing to glycemic regulation and bile acid excretion. Furthermore, these fiber fractions have demonstrated inhibitory activities against α-amylase and α-glucosidase, with reported IC_50_ values of 1.41 and 1.96 mg/mL, respectively [123,124,125].

Protein-rich sea buckthorn seed meal has also emerged as a promising source of functional ingredients and bioactive peptides. Following oil extraction, the residual seed meal may contain approximately 40–70% crude protein, depending on processing conditions and cultivar differences [126]. Enzymatic hydrolysis of these proteins can produce peptide fractions exhibiting diverse biological activities in vitro, including antioxidant, angiotensin-converting enzyme (ACE)-inhibitory, α-glucosidase-inhibitory, and dipeptidyl peptidase-IV (DPP-IV)-inhibitory effects [127]. Recent studies have further demonstrated enhanced DPPH radical scavenging activity and improved iron-reducing capacity in peptide fractions derived from sea buckthorn seed meal. Collectively, these findings highlight the considerable potential of sea buckthorn seed meal-derived peptides for application in antihypertensive and glycemic-control nutraceuticals, although additional in vivo studies and clinical validation remain necessary.

To enable the comprehensive utilization of sea buckthorn by-products, integrated biorefinery strategies combining supercritical CO_2_ extraction (SFE-CO_2_), pressurized liquid extraction with ethanol (PLE-EtOH), and enzyme-assisted extraction (EAE) have been increasingly recommended [128]. Such sequential processing approaches facilitate the generation of lipid-rich, phenolic-rich, protein-rich, and fiber-rich fractions for further utilization in food, nutraceutical, cosmetic, and biomaterial applications [129,130]. Future research should place greater emphasis on the establishment of standardized analytical protocols, scalable green extraction technologies, and integrated cascade-processing systems capable of maximizing the value and functionality of all sea buckthorn by-product fractions.

### 4.3. Potential Application Fields of By-Products and Industrial Feasibility

Sea buckthorn processing generates substantial quantities of seed meal, pomace, and leaves, which are traditionally discarded or relegated to low-value feed, representing significant biomass underutilization [131]. Recent analyses demonstrate that these matrices retain concentrated bioactives—including polyphenols, carotenoids, dietary fibers, and residual lipids—which exhibit potent antioxidant, anti-inflammatory, antimicrobial, and metabolic regulatory activities [132]. These properties position sea buckthorn by-products as high-value raw materials for multifaceted applications across the medical, materials, and food sectors. Medical and nutraceutical applications specifically leverage the carotenoid–flavonoid matrix in pomace and leaf extracts. These polar fractions demonstrate substantial antioxidant and anti-inflammatory capacities in cellular and preclinical models, with emerging evidence of chemopreventive potential, including the modulation of proliferation, apoptosis, and angiogenesis markers [133,134]. Unlike refined oils, these extracts provide the concentrated phenolic dosages (100–500 mg GAE/g) essential for therapeutic efficacy.

Active packaging and materials science applications leverage the antioxidant and antimicrobial properties of sea buckthorn polyphenols. Pomace and leaf extracts can be integrated into biopolymer matrices—such as chitosan, alginate, and whey protein—to fabricate functional packaging films. These materials inhibit lipid oxidation in meat and dairy products, extending shelf life and minimizing reliance on synthetic preservatives [135,136]. Moreover, leaf extracts rich in polyphenols and chlorophyll derivatives augment the oxidative stability of microencapsulated SBPO through radical scavenging and metal chelation, surpassing synthetic antioxidants in accelerated storage tests [137,138]. Furthermore, antimicrobial phenolics in pomace suppress spoilage organisms, while residual lipids and tocopherols in seed meal provide energy and antioxidant protection for livestock, offering a viable strategy to reduce veterinary antibiotic consumption.

Sea buckthorn by-products offer versatile opportunities for ingredient diversification in food and animal feed. Seed meal—which retains high-quality proteins, dietary fibers, and essential lipids post-extraction—can be incorporated into bakery products, nutritional blends, or livestock feed to boost nutrient density and enhance resource efficiency. Similarly, pomace-derived extracts can serve as potent natural antioxidants in lipid-rich food systems, providing formulation flexibility and appealing clean-label advantages over synthetic alternatives [139,140].

From an economic perspective, the feasibility of by-product valorization hinges on extraction efficiency, product yield, and the marketable value of the recovered fractions. Compared to traditional single-product processing models, integrated biorefinery strategies—which synchronize oil extraction, phenolic recovery, protein utilization, and fiber fractionation—can significantly maximize raw material utilization and increase economic returns per unit of biomass [110,141,142]. Such multistream utilization models are closely aligned with circular bioeconomy principles and sustainable industrial development strategies.

Despite these opportunities, several industrial challenges persist. Inherent variability in raw material composition—driven by genotypic factors, seasonal fluctuations, and processing methodologies—can compromise the consistency and functional quality of the final product [143,144]. Furthermore, large-scale extraction technologies must optimize the trade-off between recovery efficiency, solvent safety, and operational costs [145,146]. Securing regulatory approval for food, feed, and nutraceutical applications, alongside the standardization of quality control protocols, remains essential for widespread industrial adoption. Future development should prioritize integrated, cost-effective processing systems capable of maximizing the value of all biomass fractions while ensuring compositional stability and economic feasibility. Looking forward, integrated biorefinery approaches—sequentially optimizing oil extraction, polyphenol recovery, and fiber utilization—will be essential to position sea buckthorn as a model crop for zero-waste horticultural systems.

## 5. Conclusions and Prospects

SBO exemplifies the paradigm of matrix-specific functional nutrition. While its lipophilic fractions independently exert distinct cardiovascular, metabolic, and dermatological effects, the full therapeutic potential of sea buckthorn is fundamentally contingent upon the synergistic presence of co-extracted polar bioactives. Consequently, to harness these comprehensive benefits, future nutraceutical strategies must focus on the co-delivery of both lipid- and water-soluble constituents. Advanced colloidal delivery systems—such as oil-in-water (O/W) emulsions, microencapsulation, and liposomal platforms—offer robust pathways to safeguard these bioactives against gastrointestinal degradation while synchronizing their intestinal absorption.

Beyond formulation advancements, successfully translating these preclinical observations into clinical reality requires a coordinated, multi-disciplinary paradigm. First, descriptive compositional data must be integrated into predictive systems-biology frameworks, utilizing multi-omics (lipidomics, polyphenomics, and proteomics) to fully deconvolute complex bioactive interactions. Second, the current reliance on animal models must transition toward rigorous human clinical trials to establish definitive dose–response relationships. Finally, to ensure industrial viability, laboratory-scale processes must be scaled up into cost-effective, zero-waste green biorefinery operations. By systematically addressing these translational bottlenecks, future research will enable the development of standardized, evidence-based sea buckthorn products, driving the sustainable utilization of this valuable botanical resource.

## Figures and Tables

**Table 1:** Major bioactive compounds reported in sea buckthorn oil (SBO) and related fractions.

Bioactive Class	Representative Compounds	Main Fraction/Source	Reported Concentration	Reference
Polyunsaturated fatty acids (PUFAs)	Linoleic acid (LA), α-linolenic acid (ALA)	SBSO	Total PUFAs: 60–LA: 35–40% of total fatty acids; ALA: 25–30% of total fatty acids	[8,9,10]
Monounsaturated fatty acids (MUFAs)	Palmitoleic acid (ω-7), oleic acid	SBPO	Palmitoleic acid: 30–40%; oleic acid: 15–20% of total fatty acids	[8,11,12]
Phytosterols	β-sitosterol, campesterol, stigmasterol, Δ5-avenasterol	Primarily SBSO	SBSO: 10,000–15,000 mg/kg; SBPO: 1000–3000 mg/kg	[13,14,15]
Tocopherols (Vitamin E)	γ-tocopherol, α-tocopherol	Mainly SBSO	SBSO: 100–300 mg/kg; SBPO: 50–150 mg/kg	[16,17]
Carotenoids	β-carotene, zeaxanthin, lycopene, β-cryptoxanthin	Primarily SBPO	SBPO: 100–500 mg/kg; SBSO: 1–5 mg/kg	[16,18,19]
Residual trace phenolics	Trace flavonoids and phenolic acids	Refined SBSO/SBPO	Typically <5–10 mg/100 g	[20,21]
Phenolic compounds (by-products)	Isorhamnetin glycosides, quercetin glycosides, kaempferol derivatives, proanthocyanidins	Trace levels in refined oils; enriched in pomace and seed meal	Pomace: 100–500 mg/100 g; seed meal: 93–200 mg/100 g	[22,23]
Dietary fiber	Pectic polysaccharides, cellulose, hemicellulose	Pomace	Dietary fiber: 40–70% dry weight	[24,25,26]
Proteins and bioactive peptides	Essential amino acids, DPP-IV inhibitory peptides	Seed meal	Crude protein: 40–70% dry weight	[27,28]
Residual lipids in by-products	LA, ALA, oleic acid, tocopherols	Seed meal	Residual lipids: 10–15%	[29,30,31]

**Table 2:** Regional variation in oil content and major fatty acid composition of sea buckthorn oil (SBO) fractions reported in recent studies.

Region	Seed Oil Content (%)	Pulp Oil Content (%)	Major Fatty Acids in SBSO (%)	Major Fatty Acids in SBPO (%)	Reference
Xinjiang, China	8.2–12.5	15.3–24.8	Linoleic acid (35.6–39.8), α-linolenic acid (24.3–29.7), oleic acid (15.2–18.6)	Palmitoleic acid (30.5–39.4), palmitic acid (24.8–29.5), oleic acid (15.1–19.3)	[42]
Inner Mongolia, China	7.3–8.8	14.1–22.7	Linoleic acid (34.2–38.7), α-linolenic acid (23.5–28.4)	Palmitoleic acid (31.2–37.6), palmitic acid (23.9–28.2)	[48,49]
Qinghai, China	8.8–13.4	18.6–27.5	Linoleic acid (33.8–37.5), α-linolenic acid (22.6–27.1)	Palmitoleic acid (32.8–38.5), oleic acid (16.3–20.5)	[50]
Mongolia	12.67	13.5–21.4	Linoleic acid (36.9), α-linolenic acid (31.11), oleic acid (15.85), palmitic acid (7.13)	Palmitoleic acid (29.6–35.2), palmitic acid (24.5–28.8)	[49]
Finland	6.4–10.2	12.1–18.3	Linoleic acid (32.6–36.9), α-linolenic acid (20.3–24.8), oleic acid (18.1–22.3)	Palmitoleic acid (12.1–39.0)	[41]
Romania	8.1–12.7	14.2–22.1	Linoleic acid (34.1–38.5), α-linolenic acid (23.1–27.6)	Palmitic acid (23–40%), oleic acid (20–53%), palmitoleic acid (11–27%)	[8]
Russia (Siberian cultivars)	8.7–13.1	15.4–23.8	Linoleic acid (33.7–37.6), α-linolenic acid (22.7–26.5)	Palmitoleic acid (30.8–36.3), oleic acid (16.5–21.1)	[51]

**Table 3:** The main vitamins in SBO and their functions.

Types of Vitamins	Function	References
Vitamin A	Maintain normal visual function, the immune system, and cell growth and differentiation, and have antioxidant properties	[18]
Vitamin E	Antioxidant, maintaining the integrity and stability of cell membranes, and enhancing the activity of antioxidant enzymes in the body	[56]
Vitamin C	Antioxidant, maintaining cell function, enhancing immunity, and reducing the risk of cancer	[20,54]
Bioflavonoids	It has antioxidant, anti-inflammatory, and anti-allergic properties, enhances vitamin C activity, and improves microcirculation.	[19]
Vitamin B	Supports energy metabolism, the nervous system, and cell growth and division, maintains the body’s physiological functions, and enhances resistance	[20]
Vitamin K	Prevent bleeding, regulate bone metabolism, and reduce the risk of cardiovascular diseases	[19]

## Data Availability

This study is based on an integrative review of published academic and industry literature. No primary datasets were generated or analyzed in this study. All sources used are cited in the article.

## Abbreviations

The following abbreviations are used in this manuscript:

SBO	Sea buckthorn oil
SBSO	Sea buckthorn seed oil
SBPO	Sea buckthorn pulp oil
PUFA	Polyunsaturated fatty acid
MUFA	Monounsaturated fatty acid
TAG	Triacylglycerol
PA	Palmitic acid
POA	Palmitoleic acid
TNF-α	Tumor necrosis factor alpha
OA	Oleic acid
BCMO1	β-carotene 15,15′-monooxygenase
IL-1β	Interleukin-1β
IL-6	Interleukin-6
NF-κB	Nuclear factor-kappa B
LDL	Low Density Lipoprotein
NO	Nitric oxide
ACE	Angiotensin-converting enzyme
COX-2	Cyclooxygenase-2
ALA	Alpha-linolenic acid
MDA	Malondialdehyde
DF	Dietary fiber
